# Supplementary material for: T-Plastin reinforces membrane protrusions to bridge matrix gaps during cell migration
Source: Nat Commun. 2020 Sep 23;11:4818. doi: 10.1038/s41467-020-18586-3 (PMC7511357; doi:10.1038/s41467-020-18586-3)
Supplement: Supplementary file 1 — Supplementary Information [file 41467_2020_18586_MOESM1_ESM.pdf]

Supplementary Materials for

T-Plastin reinforces membrane protrusions to bridge matrix gaps during cell migration

Damien Garbett, Anjali Bisaria, Changsong Yang, Dannielle G. McCarthy, Arnold Hayer, W.E. Moerner, Tatyana Svitkina, and Tobias Meyer
Correspondence to: dgarbett@stanford.edu; tobias1@stanford.edu

This PDF file includes:

Figs. S1 to S8
Supplementary Table 1
Uncropped Western Blots

Other Supplementary Materials for this manuscript include the following:

Movies S1 to S8

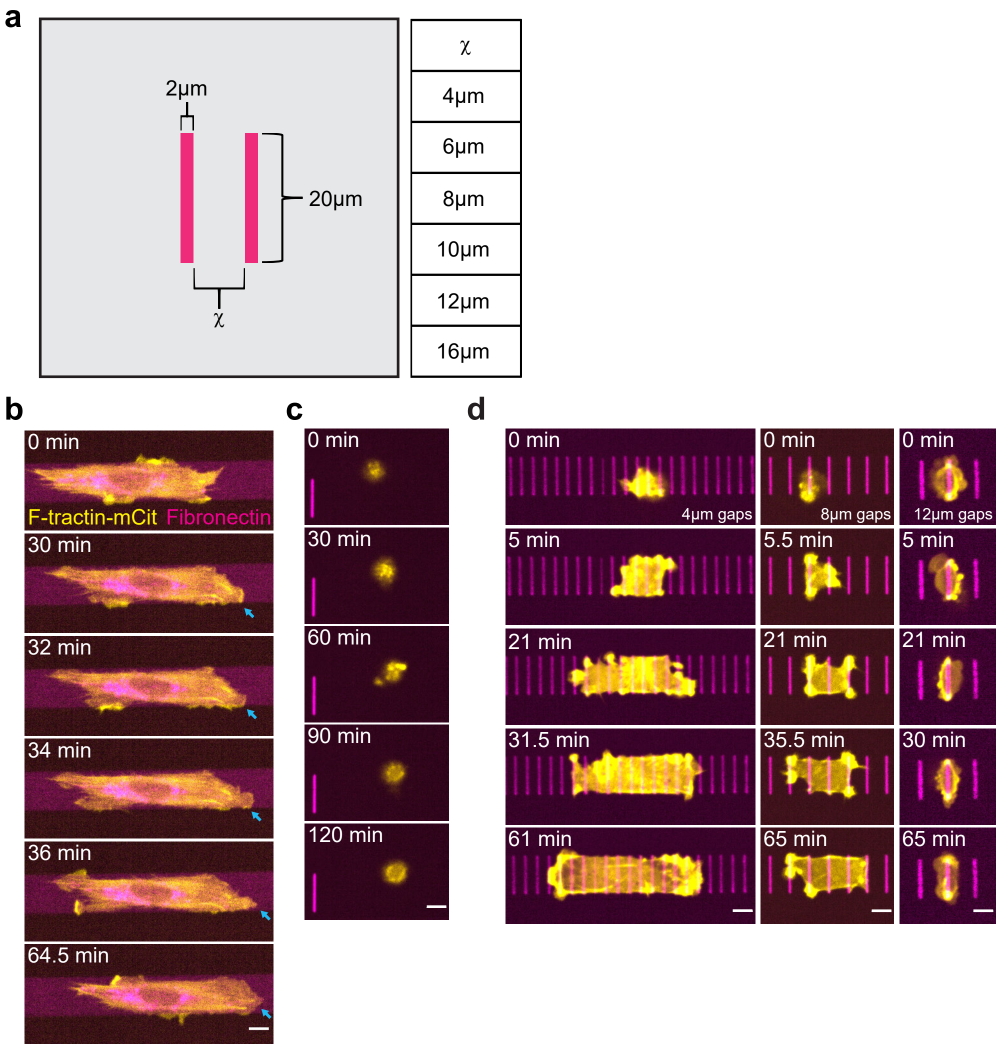

Fig. S1.

Micropattern design for adhesive gaps and solid stripes control. a Design of patterns of fibronectin (magenta) 2 μm x 20 μm stripes separated by non-adhesive PLL-PEG (gray) spaces of varying size (χ). The patterns repeat across the field of view shown in Fig. 1c. **b** HUVEC expressing F-tractin-mCitrine (yellow) were added to solid stripes of fibronectin (magenta) without gaps. Lamellipodia are highlighted by cyan arrows. **c** Similar to (**b**), but a region devoid of fibronectin, coated with PLL-PEG is shown. Cells fail to adhere and spread in these PLL-PEG regions. **d** Further examples of WT HUVEC on fibronectin ladder patterns with gaps of 4, 8, or 12 μm, similar to those shown in Fig. 1f. Bars, 10 μm.

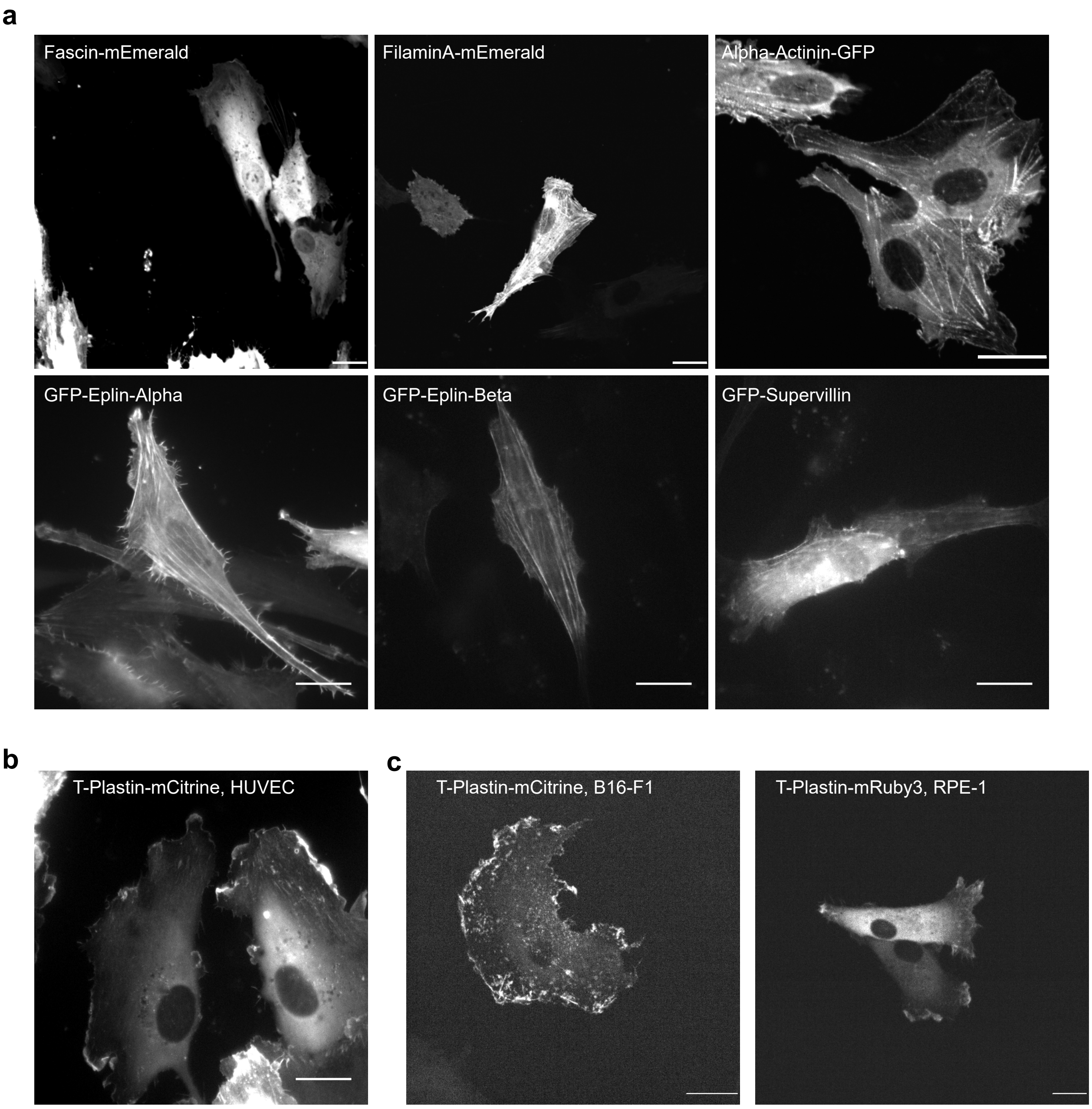

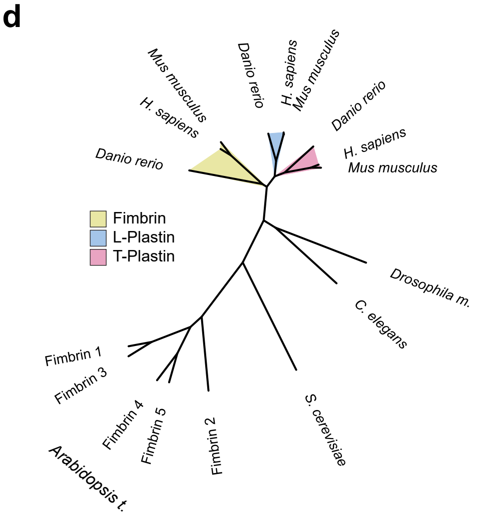

Fig. S2.

Actin bundling protein localizations and Plastin conservation. a HUVEC were transfected with the corresponding fluorescently-tagged actin bundling proteins and imaged. **b,c** Fluorescently-tagged T-Plastin was expressed in HUVEC, and in B16-F1 and RPE-1 cells. T-Plastin showed high enrichment in broad lamellipodial protrusions. Bars, 20 µm. **d** Arabidopsis have 5 plastin isoforms, yeast and invertebrates have only one. Vertebrates have three isoforms that are more similar across species than to each other and have distinct tissue distributions. T-Plastin's closest family member is L-Plastin.

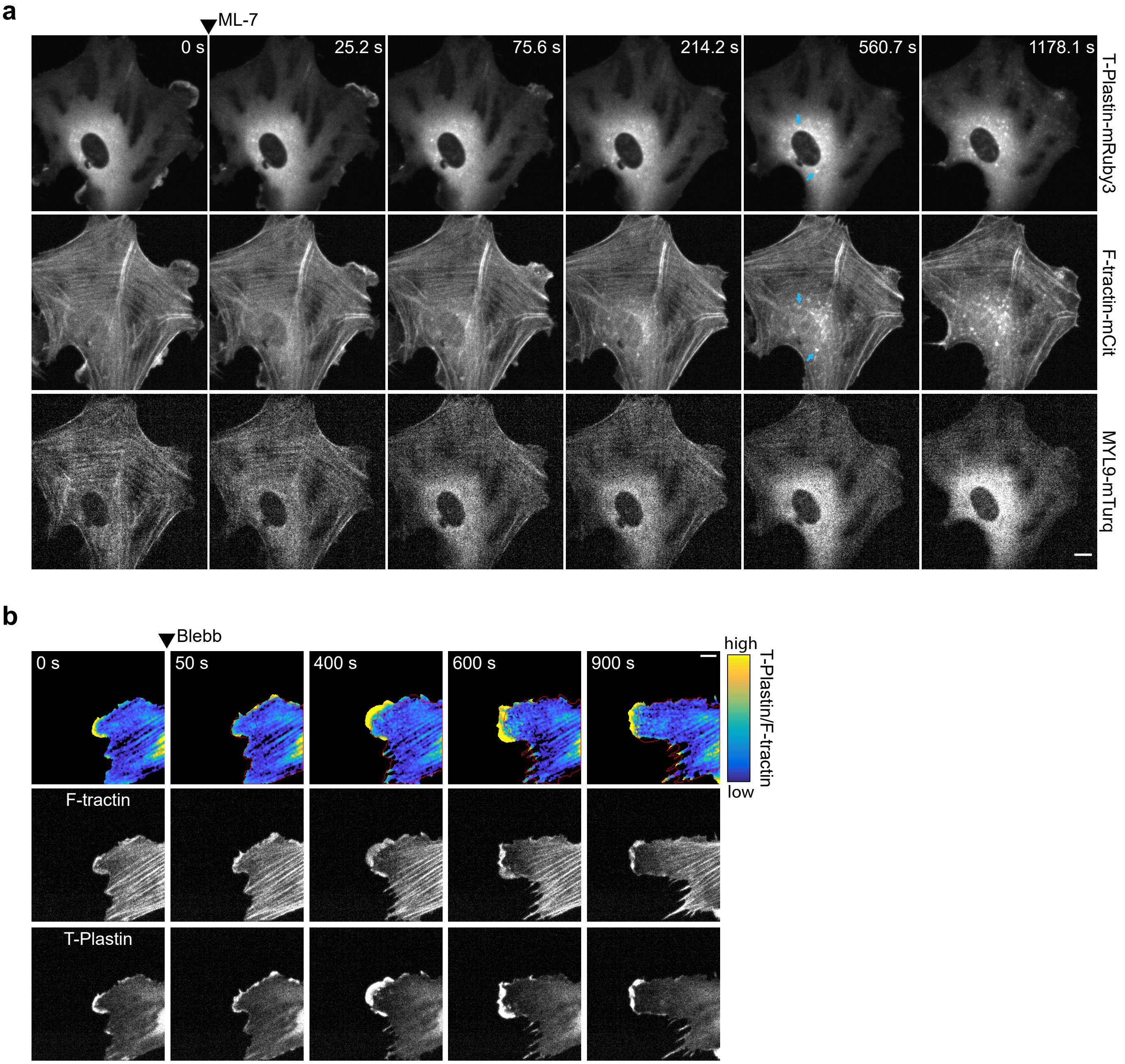

Fig. S3.

T-Plastin dynamics in sparsely plated HUVEC. a HUVEC expressing T-Plastin-mRuby3, F-tractin-mCit, and MYL9-mTurq were treated with ML-7 where indicated. MYL9 became more diffuse after ML-7 treatment and small F-actin puncta formed in the cell (cyan arrows) that were also enriched in T-Plastin. **b** HUVEC expressing T-Plastin-mRuby3 and F-tractin-mCit were treated with blebbistatin (Blebb) where indicated. Protrusions became deeper and persisted further away from the ends of actin stress fibers, however T-Plastin localization remains unchanged and associated with the leading edge. Parula colormap shows high enrichment of T-Plastin over F-tractin as yellow (99th percentile) and low as blue (3rd percentile). Bars, 10 μm.

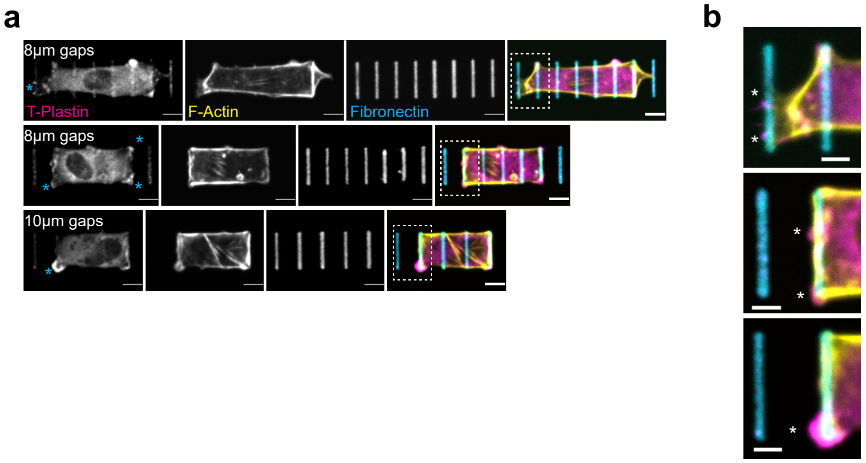

c

siRNA-1	5' GAAAGAACCUUCCGUAACU
siRNA-2	5' GAAGAGAGCUGAGAGUAUG
siRNA-3	5' GAACAUUGGUGCAGAAGAU
siRNA-4	5' GAAUUAAAGUUCCUGUUGA
sgRNA-1 (KO-1,KO-2)	5' GAAGCTAATATGCCATTACC
sgRNA-2 (KO-3)	5' AACTCATGCTGGATGGTGAC

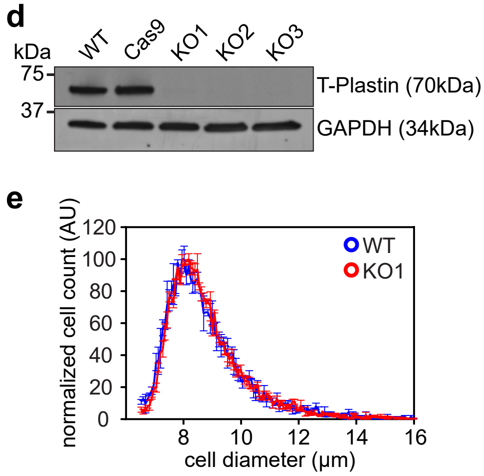

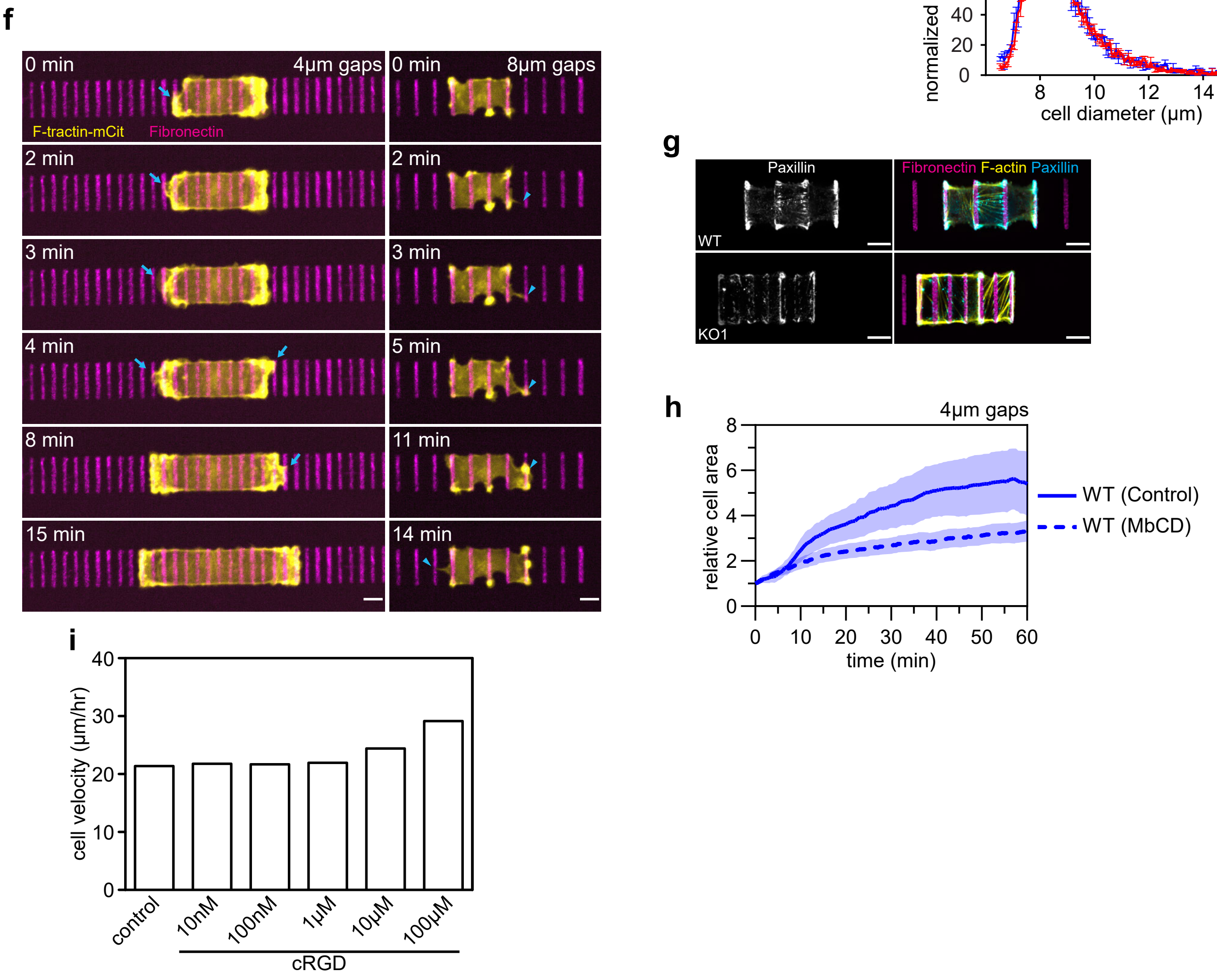

Fig. S4.

T-Plastin localization and knockout characterization on fibronectin ladder patterns. a WT HUVEC were added to fibronectin ladder patterns (cyan), fixed after 4 hrs, and stained for T-Plastin (magenta) and F-actin (yellow). Endogenous T-Plastin localized to protrusions (cyan asterisks). The white dotted boxes indicate areas blown up in (**b**). Bars, 10 µm. **b** Magnified areas from (**a**), white asterisks mark T-Plastin-labelled protrusions. Bars, 5 µm. **c** siRNA and sgRNA sequences targeting human T-Plastin used in this study. **d** Western blot of WT HUVEC and stable T-Plastin knockout (KO) CRISPR lines and Cas9 alone as control. **e** WT (blue) and KO1 (red) HUVEC were trypsinized and both cell size and number were measured in a coulter cytometer. Solid lines represent the means, error bars represent SD, n=2 independent replicates. **f** Examples of T-Plastin KO cell behavior on fibronectin ladder patterns. KO cells still form lamellipodial (cyan arrows) and filopodial (cyan arrowheads) protrusions when bridging gaps of 4 or 8 µm, respectively. Bars, 10 µm. For further examples see movies S6 and 7. **g** Further examples of focal adhesion formation of WT and KO HUVEC plated on fibronectin ladder patterns, similar to those shown in Figures 1h, 5b. Bars, 10 µm. **h** The cell area relative to the initial cell area upon first contact with a fibronectin stripe was quantified over a 1 hr period in untreated HUVEC (solid, n = 17 cells) or those treated with MbCD (dashed, n = 68 cells) used in Fig. 5e; taken from ≥3 independent replicates. Means are shown as solid lines, transparent regions indicate the 95% confidence intervals. **i** Quantification of average single cell velocities from scratch assay of HUVEC that were treated with indicated dosages of cRGD, each condition represents 24 technical replicates, of which the mean is shown.

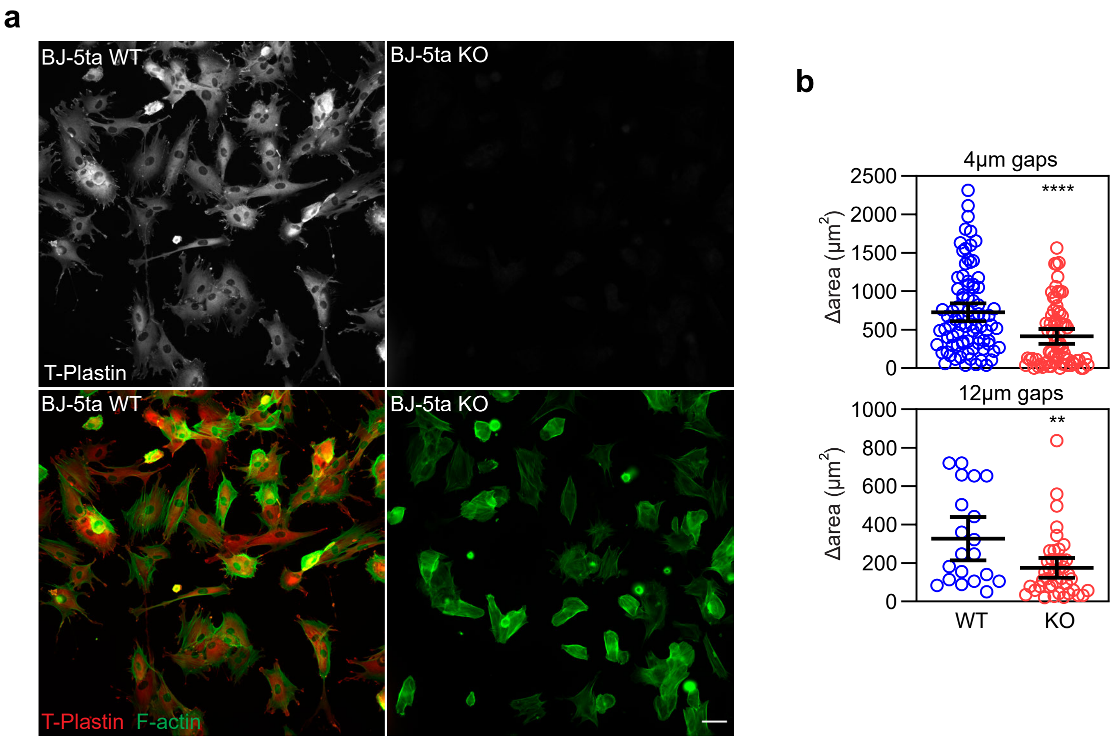

Fig. S5.

T-Plastin KO also reduces ECM gap bridging ability in BJ-5ta fibroblasts. a WT and T-Plastin KO BJ-5ta cells stained for F-actin (green) and T-Plastin (red). Bar, 50 µm. **b** Quantification of cell area change of WT (n cells for 4µm=84 12µm=20) and KO (n cells 4µm=72 12µm=41) BJ-5ta cells added to fibronectin ladder patterns with gaps of 4 and 12 µm after 1 hr; taken from ≥3 independent replicates. Black lines indicate means and 95% confidence intervals. ** P <0.01, **** P<0.0001 analyzed using a one-way ANOVA with Sidak's multiple comparison test.

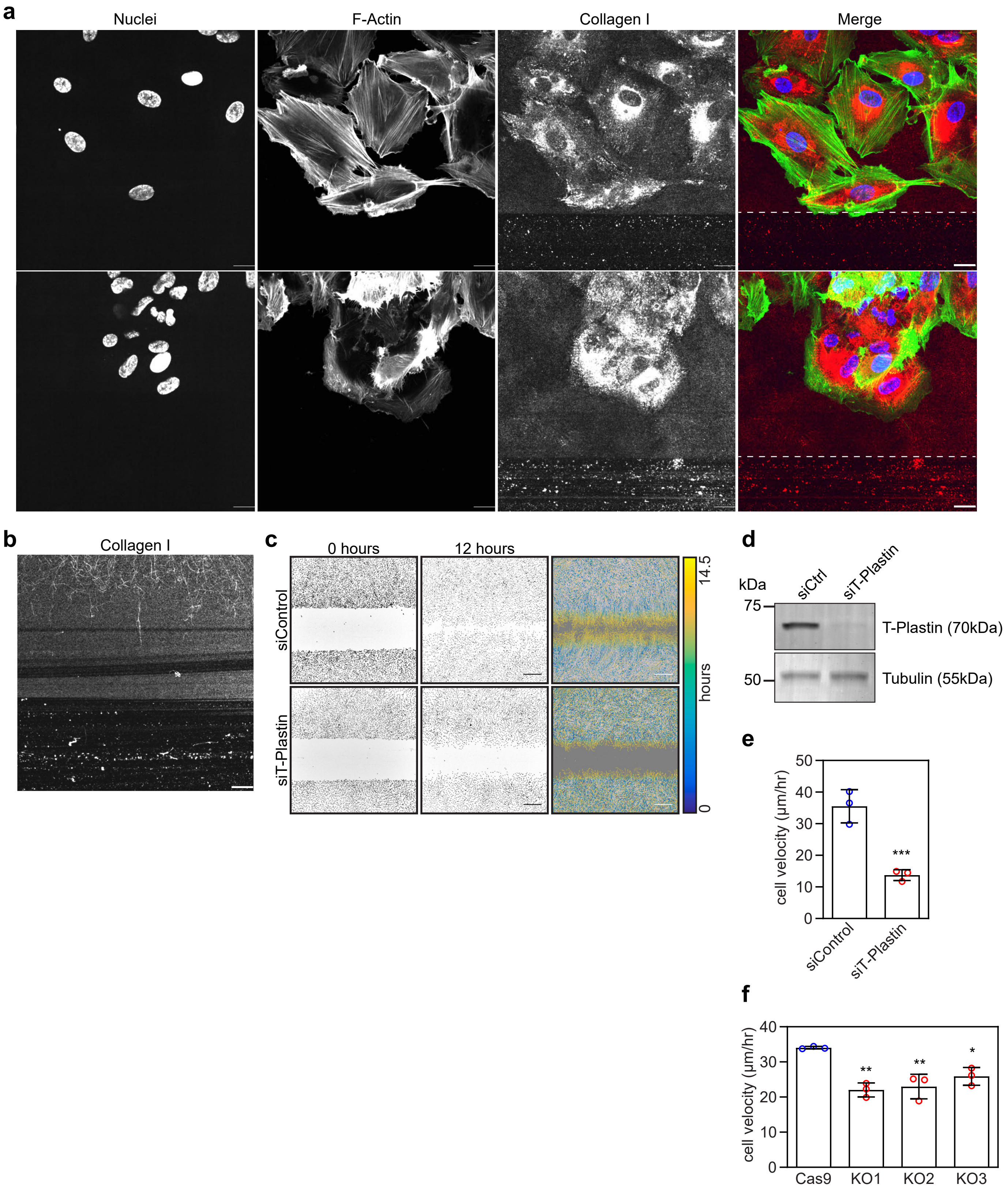
a
Nuclei
F-Actin
Collagen I
Merge
b
Collagen I
c
0 hours
12 hours
siControl
siT-Plastin
14.5
hours
0
d
kDa
siCtrl
siT-Plastin
75
50
T-Plastin (70kDa)
Tubulin (55kDa)
e
cell velocity (μm/hr)
50
40
30
20
10
0
***
siControl
siT-Plastin
f
cell velocity (μm/hr)
40
30
20
10
0
**
**
*
Cas9
KO1
KO2
KO3

Fig. S6.

T-Plastin is required for efficient directed cell migration. a HUVEC monolayers plated on uniform collagen and scratched and stained for nuclei (blue), F-actin (green), and collagen I (red). Two examples are shown, dotted white lines indicate area were collagen becomes scratched, bars 20 µm. Since these types of scratch assays are common, we wanted to illustrate that they remove the underlying ECM and cells are likely required to navigate and remodel the matrix as they migrate into the scratch. **b** Similar to (**a**), but no cells were added, bar 20 µm. Control showing collagen is removed by scratch independent of cells being removed. **c** HUVEC monolayers plated on uniform collagen were treated with siRNA targeting T-Plastin or a non-targeting control. A scratch was made and cells stained with Hoechst were imaged for 14.5 hours. A parula time composite of the nuclear masks is also shown to illustrate cell movement into the scratch with blue showing time 0 and yellow 14.5 hours respectively, bars 500 µm. **d** Western blot of HUVEC treated with control or T-Plastin siRNA probed for T-Plastin and Tubulin as a control. Please note the full uncropped western blot is shown in the supplementary material. **e** The average single cell velocities quantified from (**c**). Data is presented as the mean, empty circles represent independent biological replicates (n=3 replicates). **f** Quantification of similar scratch migration assay of cells expressing Cas9 alone or three different T-Plastin KO HUVEC lines generated. Mean is shown as bar, empty circles represent independent biological replicates (n=3 replicates). Error bars represent SD. * $P<0.05$, ** $P<0.01$, *** $P<0.001$, analyzed using a one-way ANOVA with Sidak's multiple comparison test or two-tailed unpaired t test.

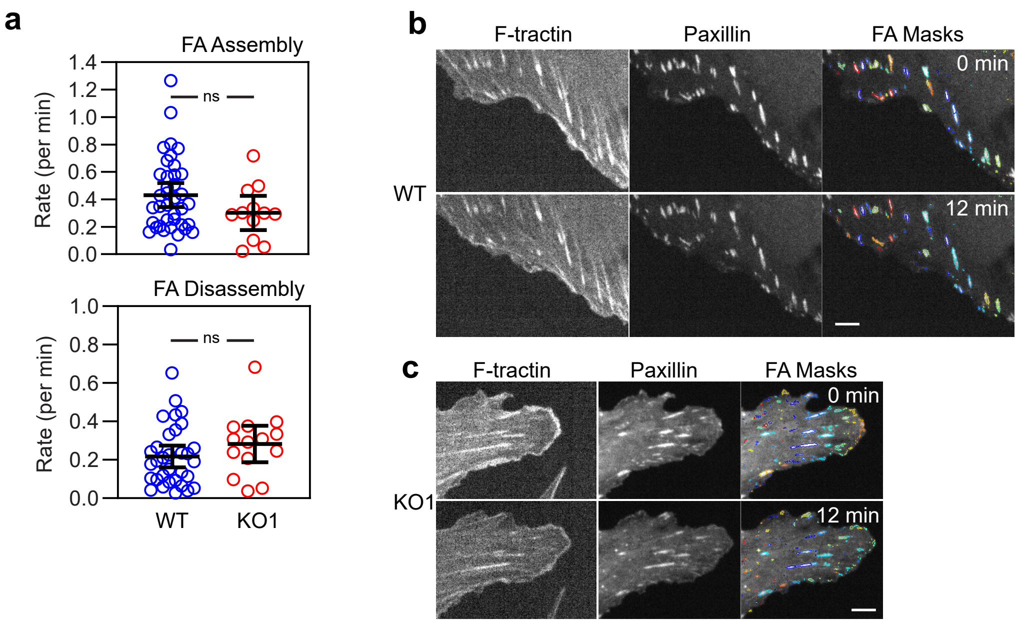

Fig S7.

Loss of T-Plastin does not alter focal adhesion kinetics. a Top, assembly rates of focal adhesions marked with mCherry-Paxillin (n adhesions for WT=38, KO1=12) Bottom, disassembly rates of focal adhesions also marked by mCherry-Paxillin. (n adhesions for WT=32, KO1=14). T-Plastin KO HUVEC (red) show no significant change in focal adhesion assembly or disassembly rates compared to WT (blue). Each dot represents an individual adhesion measured across more than 5 protrusions per condition, black bars represent the mean and 95% confidence intervals. taken from ≥3 independent replicates. Analyzed using a two-tailed unpaired t-test, ns=not significant. **b** Example images of protrusions in WT HUVEC expressing F-tractin-mCitrine and mCherry-Paxillin at 0 and 12 min. **c** Similar to (**b**), but with T-Plastin KO cells. Focal Adhesion masks used for analysis as in (**a**) are shown as colored outlines. Bars, 5 µm.

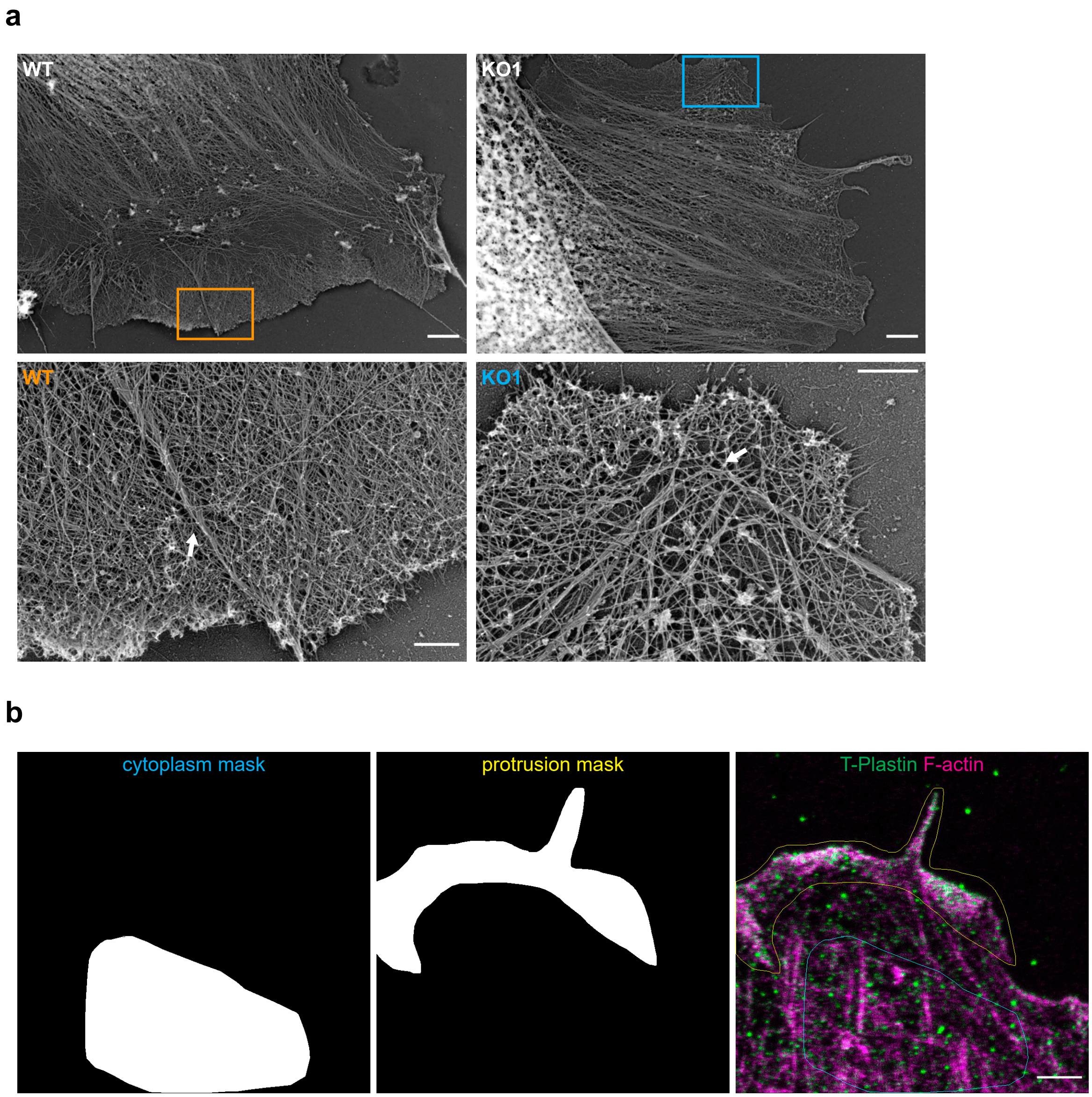

Fig. S8.

T-Plastin effects on protrusion cytoskeletal ultrastructure. a Additional examples of WT and KO HUVEC. Orange and blue boxes represent magnified areas shown below. Bars, 2 μm and 500 nm respectively. White arrows indicate filopodia, which appear straight in WT cells, but were more often bent in T-Plastin KO cells. **b** Example of masked regions used for ICQ colocalization analysis shown in Fig. 7d. Bar, 2 μm.

Supplementary Table 1. Primers used in this study.

Plasmid	Primer Name	Primer Sequence (5'-->3)'	Use
pLV-T-Plastin-mRuby3-IRES-Blast	PLS3-F	CTTCCATTTCAGGTGTCGTGAGGATCTCGCCACCATGGATGAGATGGCTACCAC	PCR
	PLS3-R	TGGATCCCCTCCTGCTCCTGCTCCTGCTCCTGCTCCCACTCTCTTCATTCCCCTGCCCATC	PCR
	mRubyF	GAGCAGGAGCAGGAGCAGGAGCAGGAGGGGATCCAATGGTGTCTAAGGGCGAAGAGC	PCR
	mRubyR	GGCCCGTCGACTCTAGAGCGGCCGCCCTCGAGGAATTTTACTTGTACAGCTCGTCCATGC	PCR
	pIRES23	ACACCGGCCTTATTCCAA	sequencing
	EF1forward	TCAAGCCTCAGACAGTGGTTC	sequencing
	PLS3-mSeq2	GAACGTTGAGTGAAGCTGG	sequencing
pLV-T-Plastin-mRuby3-p2a-MYL9-mTurquoise-IRES-Blast	PLS3-F	CTTCCATTTCAGGTGTCGTGAGGATCTCGCCACCATGGATGAGATGGCTACCAC	PCR
	PLS3RbyP2a-R	AAGTTCGTGGCTCCGGATCCCTTGTACAGCTCGTCCATGCC	PCR
	PLS3-mSeq2	GAACGTTGAGTGAAGCTGG	sequencing
	EF1forward	TCAAGCCTCAGACAGTGGTTC	sequencing
pLV-F-tractin-mRuby3-p2a-PAGFP-βactin-IRES-Neo	LV-Ftractin-F2	CTTCCATTTCAGGTGTCGTGAGGATCTCGCCACCATGGGCATGGCGCGAC	PCR
	mRuby3-P2A-R	AGGACCGGGGTTTTCTTCCACGTCTCCTGCTTGCTTTAACAGAGAGAAGTTCGTGGCTCCGGATCCCTTGTACAGCTCGTCCATGCC	PCR
	p2aPAGFP-F1	TGGAAGAAAACCCCGGTCCTATGGTGAGCAAGGGCGAG	PCR
	bActin-R1	GCGGCCGCCCTCGAGGAATTCTAGAAGCATTTGCGGTGGAC	PCR
	PAGFP-R1	TCGAGATCTGAGTCCGGACTTGTACAGCTCGTCCATGCCGAG	sequencing
	pIRES23	ACACCGGCCTTATTCCAA	sequencing
	LV-Actin-F1	TCAGATCTCGAGCTATGGATGATGATATCGCC	sequencing
pLV-RaichuCdc42-IRES-Blast	Cdc42-Rac-EV-F	CGAAGGAATGAACGAACTCTATCTCGAGAAAGAGAAAGAGCGGCCAGAG	PCR
	CAAX-EF1a-R	CGCCTCCCCTACCCGGTAGAATTCCGGCCCTCGACTTACATAATTACAC	PCR
	pIRES23	ACACCGGCCTTATTCCAA	sequencing

	EF1forward	TCAAGCCTCAGACAGTGGTTC	sequencing
pLV-Ftractin-mCitrine	Ftractin-F1	GTGTCGTGAACACGCTACCGGTCTCGAGACCATGGGCATGGCGCGACCAC	PCR
	Ftractin-R1	GATCTAGAGTCGCGGCCGCTTACTTGTACAGCTCGTCCATGCC	PCR
	pIRES23	ACACCGGCCTTATTCCAA	sequencing
	EF1forward	TCAAGCCTCAGACAGTGGTTC	sequencing
Lenti-PLS3sgRNA1	PLS3sgRNA1F	CACCGAAGCTAATATGCCATTACC	Guide ligation
	PLS3sgRNA1R	AAACGGTAATGGCATATTAGCTTC	Guide ligation
Lenti-PLS3sgRNA2	PLS3sgRNA2F	CACCAACTCATGCTGGATGGTGAC	Guide ligation
	PLS3sgRNA2R	AAACGTCACCATCCAGCATGAGTT	Guide ligation

Uncropped Westerns from Supplemental Figure 4d (T-Plastin CRISPR KO)

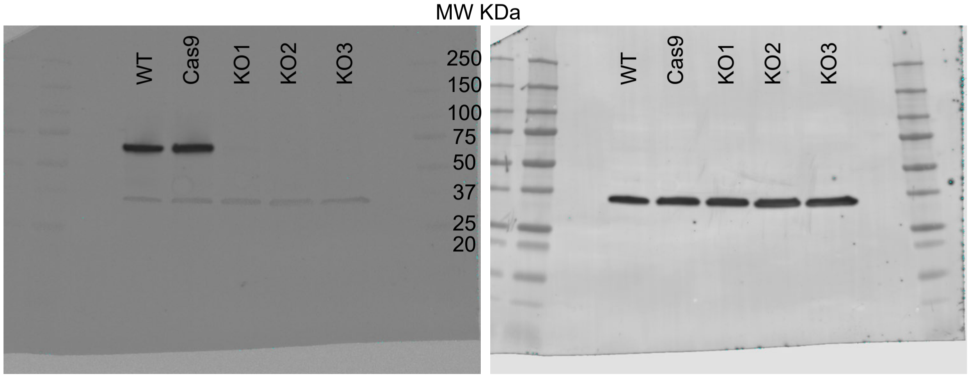

T-Plastin blot GAPDH blot

Uncropped Westerns from Supplemental Figure 6d (T-Plastin siRNA)

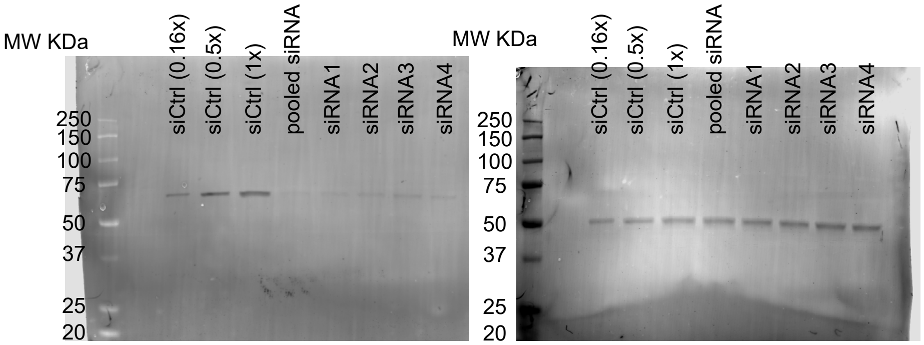

T-Plastin blot Tubulin blot
