# Supplementary material for: T-Plastin reinforces membrane protrusions to bridge matrix gaps during cell migration
Source: Nat Commun. 2020 Sep 23;11:4818. doi: 10.1038/s41467-020-18586-3 (PMC7511357; doi:10.1038/s41467-020-18586-3)
Supplement: Supplementary file 3 — Description of Additional Supplementary Files [file 41467_2020_18586_MOESM3_ESM.pdf]

Description of Additional Supplementary Files

File Name: Supplementary Movie 1
Description: HUVEC expressing F-tractin-mCitrine (yellow) on 4µm spaced labelled fibronectin (magenta) patterns were imaged every 30 seconds, corresponds to cell shown in Fig 1F. Scale bar 10 µm.

File Name: Supplementary Movie 2
Description: HUVEC expressing F-tractin-mCitrine (yellow) on 8µm spaced labelled fibronectin (magenta) patterns were imaged every 30 seconds, corresponds to cell shown in Fig 1F. Scale bar 10 µm.

File Name: Supplementary Movie 3
Description: HUVEC expressing F-tractin-mCitrine (yellow) on 10µm spaced labelled fibronectin (magenta) patterns were imaged every 30 seconds, corresponds to cell shown in Fig 1F. Scale bar 10 µm.

File Name: Supplementary Movie 4
Description: HUVEC expressing T-Plastin-mRuby3 and F-tractin-mCitrine were sparsely plated on collagen and imaged every 5 seconds. The ratio of T-Plastin divided by F-tractin is displayed as a parula colormap where high and low levels of T-Plastin compared to F-tractin are represented by yellow and blue respectively. Corresponds to Fig 4A. Scale bar 10 µm.

File Name: Supplementary Movie 5
Description: HUVEC expressing T-Plastin-mRuby3 and F-tractin-mCitrine were plated on labelled fibronectin stripe patterns spaced 4 µm apart and imaged every 30 seconds. The ratio of T-Plastin divided by F-tractin is displayed as a parula colormap where high and low levels of T-Plastin compared to F-tractin are represented by yellow and blue respectively. Corresponds to Fig 5A. Scale bar 10 µm.

File Name: Supplementary Movie 6
Description: T-Plastin KO HUVEC expressing F-tractin-mCitrine (yellow) on 4 µm spaced labelled fibronectin (magenta) patterns were imaged every 30 seconds. Corresponds to cell shown in Fig S4F. Scale bar 10 µm.

File Name: Supplementary Movie 7
Description: T-Plastin KO HUVEC expressing F-tractin-mCitrine (yellow) on 8 µm spaced labelled fibronectin (magenta) patterns were imaged every 30 seconds. Corresponds to cell shown in Fig S4F. Scale bar 10 µm.

File Name: Supplementary Movie 8
Description: HUVEC expressing T-Plastin-mRuby3 were sparsely plated on collagen and imaged every 5 seconds. An equal volume of deionized water was added to the imaging media after ~ 1min to induce hypoosmotic conditions and is indicated (+ddH2O). Scale bar 10 µm.
